# Towards X-ray free endovascular interventions – using HoloLens for on-line holographic visualisation

**DOI:** 10.1049/htl.2017.0061

**Published:** 2017-09-14

**Authors:** Ivo Kuhlemann, Markus Kleemann, Philipp Jauer, Achim Schweikard, Floris Ernst

**Affiliations:** 1 Institute for Robotics and Cognitive Systems, University of Lübeck, Ratzeburger Allee 160, Lübeck 23562, Germany; 2 Department of Vascular Surgery, University Hospital Schleswig-Holstein, Ratzeburger Allee 160, Lübeck 23562, Germany

**Keywords:** computerised tomography, holography, diagnostic radiography, magnetic tracking system, computed tomography, two-dimensional X-ray images, on-line holographic visualisation, HoloLens, X-ray free endovascular interventions

## Abstract

A major challenge during endovascular interventions is visualising the position and orientation of the catheter being inserted. This is typically achieved by intermittent X-ray imaging. Since the radiation exposure to the surgeon is considerable, it is desirable to reduce X-ray exposure to the bare minimum needed. Additionally, transferring two-dimensional (2D) X-ray images to 3D locations is challenging. The authors present the development of a real-time navigation framework, which allows a 3D holographic view of the vascular system without any need of radiation. They extract the patient's surface and vascular tree from pre-operative computed tomography data and register it to the patient using a magnetic tracking system. The system was evaluated on an anthropomorphic full-body phantom by experienced clinicians using a four-point questionnaire. The average score of the system (maximum of 20) was found to be 17.5. The authors’ approach shows great potential to improve the workflow for endovascular procedures, by simultaneously reducing X-ray exposure. It will also improve the learning curve and help novices to more quickly master the required skills.

## 1 Introduction

Over the last two decades, interventional endovascular stenting of aortic aneurysm has progressed from being a single-centre intervention to a standard procedure in many countries [1, 2]. One integral part for the success of this minimally invasive procedure is innovative and improved vascular imaging. One of the largest difficulties in learning and performing this interventional therapy is the fact that the vascular surgeon has to mentally overlay the three-dimensional (3D) vascular tree with the 2D angiographic scene. While endovascular techniques are improving, imaging during the procedure is still dependent on contrast agents and X-rays with their known disadvantages. Looking at actual development trends towards radiation free localisation of endovascular tools, the visualisation and proper integration of this spatial information will become a key technology [3]. Our work is an integral part of the development of a novel and fully X-ray free approach to perform these interventions. Even though sparing the patient is an important goal, it is even more important to reduce the surgeon's exposure to ionising radiation.

### 1.1 Related work

Even though virtual reality (VR) and augmented reality (AR) in vascular surgery [4] or neurosurgery [5] has been under active research for some time, there has – so far – not been a device that is as advanced as Microsoft's recently released HoloLens. Even though it has been available commercially only for about 1 year, there are already some results where it is applied to fluorescence-based image guided surgery [6]. Since [7] focuses on electromagnetic tracking and navigation, and not on visualisation and surgical workflow optimisation using HoloLens, the work does not stand in a competing relation. At the same time, electromagnetic tracking could be replaced by other tracking technologies, e.g. optical-fibre-based approaches [8]. The work of Parrini *et al.* [9] is based on a head-mounted display which uses two RGB cameras and pre-generated virtual objects to create a mixed-reality view and should therefore be considered as VR rather than AR. Another alternative AR device, Google Glass, has also been investigated extensively, and has, for example, been used for monitoring electromyographical responses during spine surgery [10]. However, we believe that 3D holographic visualisation is a disruptive technology that by itself has the potential to outperform every AR and VR solution.

## 2 Material and methods

**Fig. 1:**
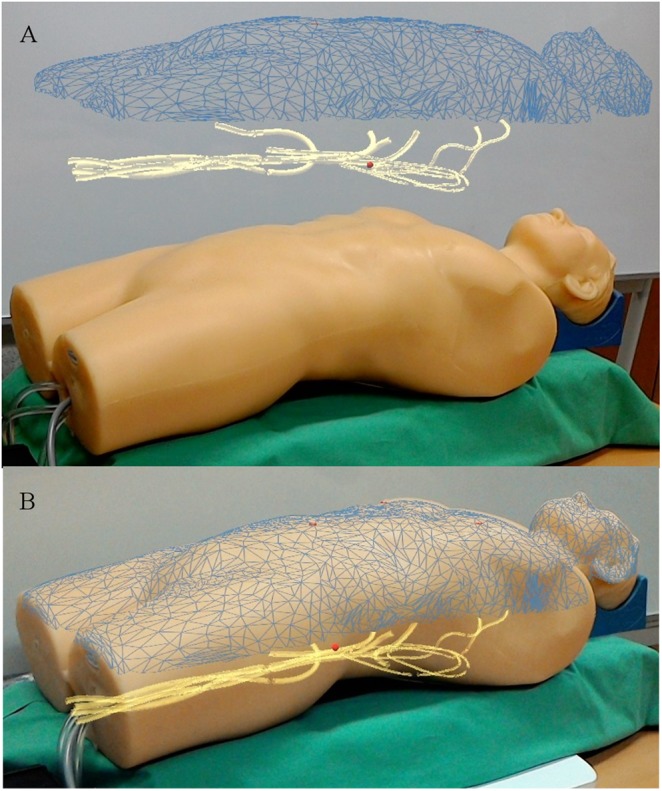
AR view of the phantom and the registered surface mesh as extracted from the CT scan (blue). The phantom's vessel tree (yellow) and the landmarks used for registration (red crosses) are clearly visible. The position of the catheter inside the vessels is visualised as a red sphere. This image can be obtained without the application of X-rays or contrast agent. The additional visual information can be rendered *a* Separated *b* Overlaid

**Fig. 2:**
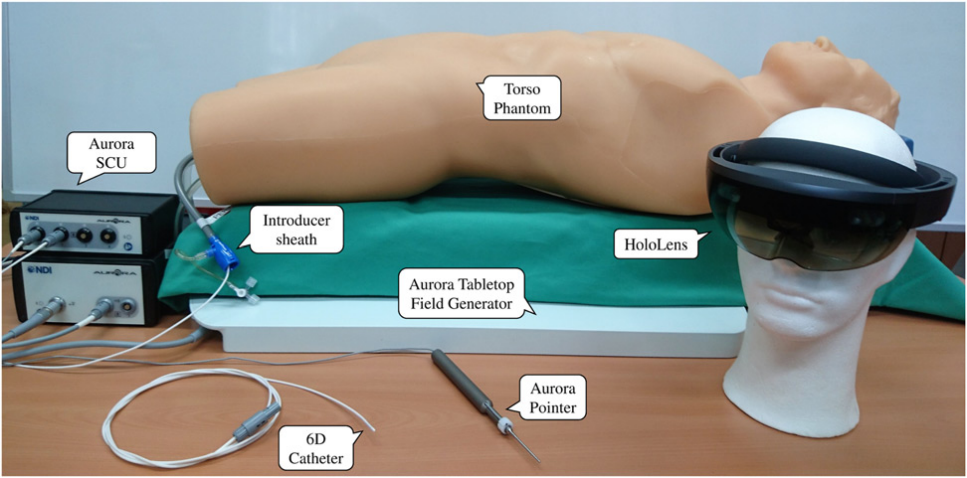
Experimental setup with all components

We report the development of a real-time navigation software, which allows a 3D view of the vascular system without any need of radiation. We used a vascular phantom model (Blue phantom FAST Trauma Full Torso Ultrasound Training Model) and an AR headset (Microsoft HoloLens) to display the vascular structures in the field of view of the surgeon. Using a landmark-based surface registration of a computed tomography (CT) scan and marching cubes segmentation of the vessel tree, it is possible to visualise both the surface and the vessels in the AR display as shown in Fig. 1. Using a magnetic tracking system (i.e. Northern Digital's Aurora), we can also display the position and orientation of a catheter inside the vessels. The complete experimental setup and all components are shown in Fig. 2. The system is developed using Unity and C# to display the rendered 3D structures extracted from a planning CT scan. Furthermore, using a middleware [11] for tracking systems running on a desktop PC (connected to HoloLens using WiFi), the position of electromagnetic markers is streamed to HoloLens in near-real time. We used NDI's Aurora V3 system, together with the flat-panel tabletop transducer, to track two magnetic markers: first, a pointer tool used for registration and second, a catheter tool which can be inserted into the phantom's vessel tree. Data is delivered with full six degree of freedom (DoF) at an update rate of 40 Hz.

### 2.1 Voice control

To further increase user acceptance and comfort, several voice commands have been implemented. They can be used to hide/show the main parts of the visualisation, like the landmarks used for registration, the mesh surface of the patient, the canvasses and the vessel tree. Furthermore, it is possible to move the vascular tree out of the patient and make it hover above the patient's surface (Fig. 1). Voice control is also used for calibration, as described below.

### 2.2 Calibrating the scene

To visualise the tracked marker positions with respect to the 3D image data, and align the whole virtual AR scene with the real world, two extrinsic calibrations are required. Both calibrations are landmark based and performed using the Horn algorithm [12]. The process has to be performed quickly and easily, such as to not interfere with the surgeon's typical workflow.

The first calibration has to align the reference coordinate system (of the CT volume data set) with the tracking base coordinate. This is achieved by selecting three to five distinct landmarks in the CT scan and extracting their coordinates. Then, the same points have to be touched in the real world with the magnetic pointing device. For each point picked with the pointer, the voice command ‘pick position’ is used to select it for calibration. After the last point has been selected, the voice command ‘calibrate tracking’ is used to perform the registration process. The second calibration step – aligning the virtual rendering of the surface and vascular tree meshes with the real world – is also done using the magnetic pointer. This time, however, it is necessary to point at the virtual landmarks displayed on the surface mesh instead of the real landmarks on the patient (or phantom). Since it is relatively difficult to accurately place the pointing tool on an exact point of the virtual scene, this calibration step is performed on an axis-by-axis basis, i.e. for each landmark, each axis of the coordinate frame is calibrated individually. Again, this is done using voice commands.

### 2.3 Additional canvasses

**Fig. 3:**
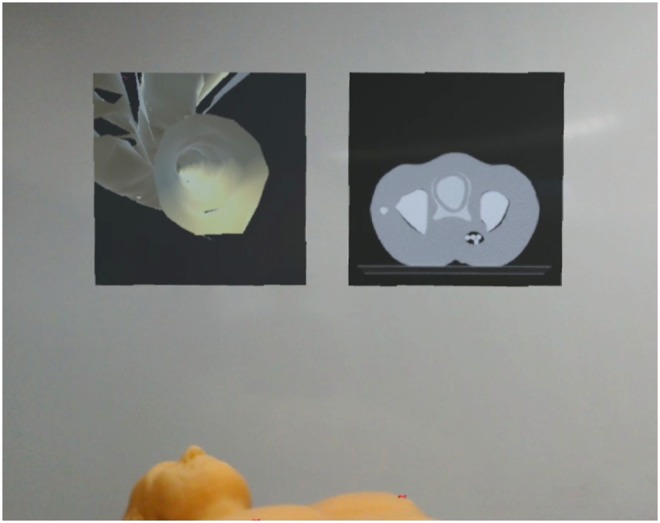
Two virtual canvasses are hovering above the patient. The left canvas shows the PoV of the catheter inside the vessel and the right canvas renders the representative CT slice in respect to the tracked catheter position

The main goal of the project is to generate an AR view that will provide the surgeon with all information required. Clearly, this does not only require an accurate 3D representation of the vessel tree, the catheter and its position, but it is also necessary to display the imaging data typically used. Consequently, we have implemented two further canvasses displaying this additional data. The first canvas shows the transverse CT slice corresponding to the current catheter position. Additionally, the catheter position can be displayed in the image data. The second additional canvas shows a point of view (PoV) perspective of a virtual camera placed on the tip of the catheter, showing what an observer inside the vascular tree would see. Both canvasses automatically rotate such as to always be perpendicular to the user's gaze direction. These canvasses are shown in Fig. 3.

### 2.4 Evaluation

To evaluate the usability and clinical significance of our work, it was presented to a panel of vascular surgeons, radiologists and thoracic surgeons with no previous experience with AR or VR settings. Each surgeon had the same task: after performing both calibration steps, the surgeons had to insert the catheter through the introducer sheath and navigate the tip through the vessels to the heart. We focused on workflow integration and visualisation quality as evaluation criteria. The following questions were asked, all ranged on a five-point Likert scale:

- Do you believe the calibration procedure could be performed like you just did in the OR? Rate from 1 (absolutely not) to 5 (definitely, not a problem).
- Is the visualisation of the vessel tree inside the phantom convincing? Rate from 1 (absolutely not) to 5 (looks like the real thing).
- Is the lag between motion of the electromagnetic marker and the virtual representation a problem? Rate from 1 (deal breaker) to 5 (not noticeable).
- Could you imagine employing this system during endovascular interventions? Rate from 1 (absolutely not) to 5 (definitely).

## 3 Results and discussion

**Fig. 4:**
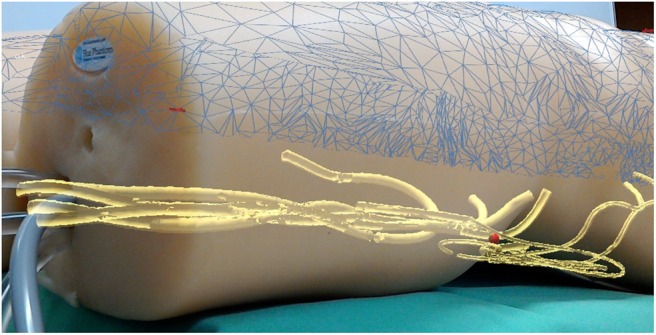
Virtual vascular tree with the tracked catheter position (red sphere) inside a vessel

With our developed framework, it is now possible to visualise the vascular tree and the tracked position of an endovascular catheter as a 3D holographic view inside the patient. Using the two proposed extrinsic landmark-based calibrations, the virtual objects are precisely aligned with the real world, resulting in a convincing holographic illusion. Fig. 4 shows a result of an AR guided vascular examination.

**Fig. 5:**
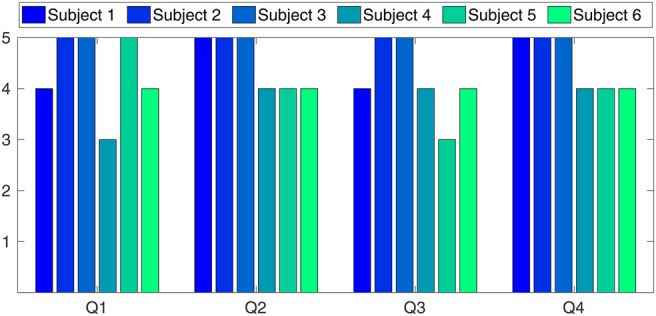
Six highly experienced surgeons have participated in a survey after testing the developed system. The overall positive validation clearly approves the high potential and the strong acceptance of the developed technology in clinical applications

HoloLens is able to display the AR data at a rate of 60 fps. Our performance tests showed that rendering the surface mesh of the phantom (10,000 triangles) and the vessel tree (50,000 triangles) did not significantly degrade the rendering performance. Unfortunately, however, a noticeable lag between physical motion of the electromagnetic markers and the marker's virtual representation was observed. We believe that this latency is caused by the WiFi connection between HoloLens and the desktop PC running the tracking middleware and, potentially, by the mismatching frame rates (40 Hz for Aurora and 60 Hz for HoloLens). The results from the technological demonstration to clinical experts and a following short survey are shown in Fig. 5. The survey was taken after each subject has experienced a complete workflow with the new AR system, including both calibration steps, and an examination of a simulated endovascular procedure. As mentioned in Section 2.4, the evaluation included four questions, where the answers were given on a five-point Likert scale. The general impression of the physicians, however, was extremely positive. All volunteers expressed their enthusiasm about the potential of the technology and the integration of AR in a medical application. The ratings clearly show the high potential and the strong acceptance of the developed technology in clinical applications. The average score (out of a maximum of 20) was 17.5.

### 3.1 Accuracy

Regarding the accuracy, the first calibration (tracked catheter to CT data) is critical for the overall precision of the system, whereas the second calibration (3D data to real world) is not – it is merely a visual cue. However, in case of an overlaid visualisation as shown in Fig. 1, the accuracy of the second calibration significantly influences the visual perception. Evaluation of the visualisation accuracy was carried out by analysing the error of the first calibration step (tracking coordinates to virtual CT). To acquire this, a full calibration was performed with four characteristic landmarks and repeated in *n* = 20 trials. The overall root-mean-square (RMS) error (point-to-point correspondence) was 4.347 mm with $\sigma = 0.709\; {\rm mm}$. Three sources of error mainly contribute to this inaccuracy in calibration. First, accuracy of the tracking system is specified by the manufacturer as 0.8 mm RMS for position and $0.7^\circ $ for rotation. In fact, these are optimal results under idealised settings. Hence, the effective accuracy of the magnetic tracking system depends on the pointing-tool design, as well as the presence of metal. Second, individual imprecisions are introduced into the system by picking the landmarks on the patient's surface with the pointer device. Finally, the main source of error is the distinction between the preoperative CT scans and the actual patient's position. Even in our idealised laboratory setup we faced the problem of non-rigid structures. This negatively affects the two calibrations, as well as the visualisation of the vessels with the tracked catheter. As a result, the catheter tip may appear outside the vessels which could be disturbing for the physician. Viable solutions are currently under development. To update the CT data with intra-operative live imaging data is a promising approach. Another even simpler solution is to simulate a virtual snapping of the rendered catheter into the vessels’ centre. The validation of the second calibration – the 3D holographic overlay visualisation – is a challenging task and requires the development of suitable validation methods which will have to be developed in future works.

### 3.2 Shortcomings of HoloLens

Unfortunately, we experienced a slight mismatch between the PoV of the HoloLens user and the streamed ‘mixed realty’ image data for creating photos and videos with external applications. It seems that, however, the calibration of the integrated RGB camera – serving as the ‘user's eyes’ – and the holographic images lacks in accuracy. However, this is only a problem for sharing the user experience (e.g. through an external monitor) and does not affect the holographic visualisation for the operator. The registration quality will be evaluated more closely during future work.

Additionally, we initially hoped that registration of the surface extracted from the CT scan to the surface mesh measured by HoloLens would be possible. It turned out, however, that even when using the highest possible meshing resolution, the surface data generated by HoloLens is far to crude to allow being used for surface registration algorithms. Methods like the iterative closest point method [13] or novel colour-enhanced registration approaches can thus not be used. Consequently, we had to rely on landmark-based registration for this initial study. It is planned to incorporate higher quality surface scanning technology, like Intel's RealSense or Microsoft's Kinect v2 cameras [14].

One problem typically stated about HoloLens – its rather limited field of view of ∼$30^\circ $ – was not noticed by any of the volunteers. It seems that this shortcoming is more obvious to technical users and of little concern to medical personnel which tends to focus their attention on a relatively small part of their visual field.

## 4 Conclusion and outlook

The visualisation of vessels, and spatial position and orientation of surgical tools during interventional endovascular stenting of aortic aneurysm has become of crucial importance for the success of this minimally invasive procedure. This Letter presents the development of a real-time navigation framework, which allows a 3D holographic view of the vascular system without any need of radiation. Using extrinsic landmark-based calibrations, the virtual objects are precisely aligned with the real world, resulting in a convincing holographic illusion. The developed system was evaluated by presenting it to a panel of experienced vascular surgeons, radiologists and thoracic surgeons. Subsequently, a survey was taken after each subject has experienced a complete workflow. The overall positive validation clearly shows the high potential and the strong acceptance of the developed technology in clinical applications. Our preliminary results of navigated virtual angioscopy are promising. The ‘virtual angioscope’ may improve intraoperative visualisation, placement of guidewires and stents. It may reduce the amount of contrast agents and exposure to X-rays. The prototype also offers the possibility of intervention planning and simulation, which in turn will lead to a reduced learning curve and therefore increased patient safety.

This was a first proof-of-concept study to assess the general acceptance of this AR approach among clinicians. In a next step, we will analyse the registration errors for the two landmark-based calibrations. Especially, the analysis of the 3D holographic overlay visualisation accuracy is a challenging task and requires the development of suitable validation methods. Furthermore, next steps are the analysis of registration inaccuracies of the RGB camera, as well as integration of a high-resolution depth camera, like Intel's RealSense or Microsoft's Kinect v2. Finally, it will be interesting to see if it is possible to replace the magnetically tracked catheter by other means of localisation, like optical fibre-based approaches [8].
